# Widespread learned predator recognition to an alien predator across populations in an amphibian species

**DOI:** 10.1038/s41598-023-41624-1

**Published:** 2023-09-05

**Authors:** Nuria Polo-Cavia, Rosa Arribas, Carlos Caballero-Díaz, Ángel Baltanás, Ivan Gomez-Mestre

**Affiliations:** 1 Department of Biology, Universidad Autónoma de Madrid. Ciudad Universitaria de Cantoblanco, 28049 Madrid, Spain; 2 Present Address: Monitoring Team on Natural Processes ICTS-RBD, Doñana Biological Station, CSIC, E-41092 Seville, Spain; 3 Department of Ecology, Universidad Autónoma de Madrid. Ciudad Universitaria de Cantoblanco, 28049 Madrid, Spain; 4 Ecology, Evolution and Development Group, Doñana Biological Station, CSIC, E-41092 Seville, Spain

**Keywords:** Behavioural ecology, Conservation biology, Evolutionary ecology, Freshwater ecology, Invasive species, Molecular ecology, Wetlands ecology

## Abstract

Alien predators are a major cause of decline and extinction of species worldwide, since native organisms are rarely equipped with specific antipredatory strategies to cope with them. However, phenotypic plasticity and learned predator recognition may help prey populations to survive novel predators. Here we examine geographical variation in the learning ability of larval spadefoot toads (*Pelobates cultripes*) to recognize invasive predatory crayfish (*Procambarus clarkii*). We compare the learning-mediated behavioural responses of tadpoles from six populations across two regions in Spain (central and southern), with different histories of exposure to the presence of the invasive species. Two of the populations showed innate recognition of chemical cues from the invasive crayfish, whereas three of them learned to recognize such cues as a threat after conditioning with conspecific alarm cues. Learning abilities did not differ among southern populations, but they did among central populations. We assessed patterns of genetic variation within and among these two regions through microsatellite markers and found low genetic divergence among the southern populations but greater differentiation among the central ones. We hypothesize that similar responses to the invasive crayfish in southern populations may have arisen from a combination of extended historical exposure to this introduced predator (~ 50 y) and higher levels of gene flow, as they inhabit a highly interconnected pond network. In contrast, populations from central Spain show lower connectivity, have been exposed to the invasive crayfish for a shorter period of time, and are more divergent in their plastic responses.

## Introduction

Alien predators are considered to be one of the major threats to global biodiversity loss, causing declines and extinction of species worldwide^1–3^. Introduced predators can create novel ecological contexts, posing new threats to which antipredatory responses of native prey may lack adaptive value^4–6^. Native prey are usually equipped with adaptive morphologies and/or behaviours to cope with local, coexisting predators. However, prey are often naïve to the hunting tactics of novel predators with whom they lack a shared evolutionary past^1, 7^. Because of this, alien predators frequently cause more severe impacts to prey populations than native predators^8^.

Despite the initial advantage of alien predators over naïve prey, prey populations enduring predator invasions may avoid extinction by adapting to the new threat^9^. Conditions favouring the persistence of local prey populations include moderate levels of predation pressure, large enough population size, the existence of sufficient genetic variation and/or phenotypic plasticity in the response to the novel predator within the population^10–12^. Adaptive variation, both genetic and phenotypic, appears then critical for native prey to evolve strategies to cope with alien predators, allowing the transition to their novel selective regimes. Nonetheless, because genetic shifts generally require multiple generations to occur, they are often too slow to keep pace with such a rapid environmental change^13^. In that light, phenotypic plasticity may be an essential mechanism for prey populations to survive novel predators, tempering the immediate impact of invasions and buying time for genetic responses to evolve, or be co-opted from defences against native predators^14–18^. In particular, behavioural plasticity –the ability of organisms to vary their behaviour in response to internal or external stimuli– constitutes the most rapid way to achieve behavioural shifts and improve individual fitness in response to human-induced environmental change exceeding the evolutionary response rate of the populations, and it may therefore represent the first line of defence for native prey to persist to introduced predators^19–22^.

Amphibians are the most threatened group of vertebrates with ca. 41% of the species endangered^23, 24^. Over the last decades, numerous studies have linked the introduction of alien predators with global amphibian declines, and even local extinctions^25–33^. Amphibian eggs and tadpoles are particularly vulnerable to alien aquatic predators, which can consume them intensively^28–31^. Naïve tadpoles, like many freshwater organisms, typically respond to the presence of water-borne cues from local predators by developing defensive morphologies and adjusting their behaviour^34, 35^ reviewed in^36, 37^. In contrast, tadpoles usually fail at inducing adaptive responses against alien predators, since activation of plastic defences necessarily requires predator recognition, and naïve tadpoles may be unable to recognize introduced predators with which they lack evolutionary history^38–42^. However, experimental studies have shown that many aquatic organisms including tadpoles can learn to recognize new predators as a threat by associating the unknown predator stimulus with conspecific alarm cues^41, 43–46^. Alarm cues are released when prey skin is damaged during a predatory attack, warning nearby individuals of imminent risk of predation and being crucial tools in associative learning^40, 47, 48^.

Hence, learning might be key to enabling the use of inducible defences against introduced predators, which can prove critical for amphibian populations until innate recognition (i.e. genetic adaptation) evolves. In this regard, tadpoles in some amphibian populations have been reported to recognize introduced predators^39, 49–52^, suggesting that, given enough time, native amphibians may evolve the ability to innately detect and avoid novel predators over generations, presumably assisted by behavioural plasticity through recurrent individual learning during the initial exposure. Geographic variation in plastic or innate responses against novel predators are likely to arise among prey populations, mostly due to variation in local predator abundance and history of exposure to the novel predators^9, 53^. Moreover, among-population variation in their behavioural plasticity and learning capacity may be influenced by available genetic diversity and gene flow with other populations with varying degrees of exposure to the novel predators. Thus, given sufficient genetic variation and selection from the novel predator, adaptive plasticity and even innate recognition can evolve locally, and even be exported to neighbouring populations with less predation pressure^54, 55^. Conversely, the evolution of such adaptations may be hindered by incoming gene flow from non-adapted populations^54, 56, 57^.

Here we compare innate responses and the ability to learn to detect chemical cues from a harmful invasive predator, the red swamp crayfish, *Procambarus clarkii* (Girard 1852), across multiple populations of the western, or Iberian, spadefoot toad, *Pelobates cultripes* (Cuvier 1829), with different histories of exposure to the presence of this invasive crayfish. Taking into account differences in the extent (abundance and history) of the invasion among toad populations and their genetic structure, we experimentally compare behavioural plasticity of tadpoles (innate and learning-mediated). According to the ‘plasticity-first’ evolution hypothesis^14–16^, the predatory pressure posed by the invasive crayfish would select for plasticity to increase in the populations in a first step, whereas plasticity would decrease in a second step when adaptive responses become fixed (i.e. the adaptation in the cue recognition system of tadpoles and the evolution of innate responses might occur at a later time). Given the short time elapsed since crayfish were first brought to the Iberian Peninsula (ca. 50 years ago), we predict that the populations are mostly in the first step (increasing plasticity). Thus, we expected learning-mediated behavioural plasticity of tadpoles to be influenced by the level of predatory impact posed by the crayfish, so that populations under stronger predatory pressure would have experienced stronger selection in favour of genotypes with greater learning ability^58–60^. We also analysed variation in the underlying genetic structure of the populations using neutral microsatellite markers to assess their genetic variation and patterns of among-population connectivity, so as to understand the population structure within which the among population differences in plasticity would have evolved.

## Methods

### Study system and study animals

The red swamp crayfish, *Procambarus clarkii*, is a worldwide invasive predator causing huge biodiversity loss in freshwater ecosystems. Native to south-eastern North America, *P. clarkii* is currently present in up to forty countries in four continents^61^. In Spain, it was introduced in the early 1970’s for commercial aquaculture purposes, in Badajoz province in 1973 and in the Lower Guadalquivir in 1974, following multiple translocations and gradually expanding its populations throughout almost the entire Iberian Peninsula^62, 63^. *Procambarus clarkii* is a voracious species with a broad trophic niche that can completely alter the trophic structure of invaded ecosystems, causing special damage to native amphibians through intense predation of eggs and tadpoles^28, 64–67^. Changes in morphology, behaviour, and life cycles of tadpoles have been observed in some amphibian populations in response to *P. clarkii*^52^, especially when crayfish cues were paired with alarm cues from attacked conspecific tadpoles^38, 64, 68, 69^. In contrast, populations of the common frog, *Pelophylax perezi*, and the western spadefoot toad, *Pelobates cultripes*, in southern Spain have consistently shown lack of innate antipredatory responses to chemical cues from the same invasive crayfish^38, 41^. Further, we have demonstrated that learned predator recognition via association with conspecific alarm cues successfully triggers antipredatory behaviour and improves survival of *P. cultripes* tadpoles in predation trials with the alien crayfish^41^.

Here we compare the ability to learn predator recognition in *P. cultripes* across six populations distributed between two regions of the Iberian Peninsula: three populations from Madrid and Segovia provinces (central Spain) –Manzanares (MAN), Colmenar (COL) and Sto. Tomé (STOME)–, and three populations from Huelva province (Doñana National Park, southern Spain) –Espajosas (ESP), Jabata (JAB) and El Llano (LLA). These populations were selected based on abundance and historical presence of invasive crayfish. The southern region around the Guadalquivir marshes was first invaded by *P. clarkii* in the 1970’s^62, 63^, whereas the central region was colonized more recently, likely by the end of the 1980’s^70^. Within each of both regions, we selected populations that in recent years have consistently shown different degrees of presence of the invasive crayfish, from ‘very abundant’ (southern Spain: ESP; central Spain: MAN) to ‘intermediate or intermittent’ presence (southern: JAB; central: COL), to ‘absence’ (southern: LLA; central: STOME). This information about crayfish abundance was based on previous fieldwork surveys in the southern region (databases from the Monitoring Program in the Doñana Natural Area, available since 2009; http://icts.ebd.csic.es/en/monitoring-program) and direct personal observations (I. Gomez-Mestre, C. Díaz-Paniagua and I. Martínez-Solano). Given the distinct histories of exposure to the invasive crayfish, and the likely (and confirmed, see below) genetic divergence between the two regions, we analysed each region independently to test for among-population divergence in antipredator responses and learning ability.

In spring 2017 we collected portions of 3–6 egg clutches in early stages of development (< 10 Gosner^71^) from each population. Eggs were transported to the Doñana Biological Station in Seville and housed in a walk-in climatic chamber to guarantee naïveté of experimental tadpoles to predator cues. Upon hatching, we raised tadpoles individually in 3 L plastic buckets with carbon-filtered dechlorinated tap water at 20 °C and 12:12 L:D photoperiod. Water was renewed twice weekly, and we subsequently fed tadpoles with ground rabbit chow and lightly boiled spinach. Additionally, we used fyke-nets to collect adult crayfish at the study populations to be used as predator cue donors. Donor crayfish were transported to the laboratory and housed individually in 3 L buckets in a climatic chamber separated from that of tadpoles to avoid chemical or visual contact with the predators prior to the experiments. Crayfish were fed spinach ad libitum, and temperature and photoperiod were the same as those of tadpoles. Crayfish and surviving tadpoles from Doñana National Park were euthanized after the experiments as indicated by the National Park authorities, whereas tadpoles from the northern populations were kept until metamorphosis and released as juveniles at their ponds of origin after standard prophylaxis procedures.

### Preparation of chemical stimuli

To prepare predator chemical cues, we filled each donor crayfish aquarium with 1.5 L of dechlorinated tap water, to be pervaded with predator cues. To avoid potential confounding effects of the diet affecting conditioning or learning responses, we used starving crayfish to provide the cues (i.e. we examined tadpole responses to predator’s signature odour^47^; see also^72^ for definitions of the main cue types involved in risk perception). We kept crayfish unfed for 4 consecutive days before placing them in the donor aquaria, to ensure that no food remained in their digestive tracts^73^. Since predator cues last approximately 2–4 days in water^74^, we waited 2 additional days and then extracted and mixed the water from five donor aquaria and froze it in 10 mL aliquots until use. Ice aliquots containing predator cues have been previously used in experiments with *P. cultripes* and other anuran species, proving that tadpoles are able to detect and respond to such cues after freezing^41, 42^. Control water was prepared following the same procedure but without placing crayfish in the aquaria^41, 42^.

Tadpole alarm cues were prepared from three conspecific donor tadpoles. Tadpoles were euthanized by immersion in a highly concentrated solution of MS-222 and homogenized with a bench top homogenizer (Miccra D-1, Germany). We then diluted the homogenate in 600 mL of carbon-filtered, dechlorinated tap water and filtrated it with filter paper to remove solid particles. The water containing the alarm cues was immediately frozen in 10 mL aliquots until use^75^.

### Behavioural plasticity

Behavioural plasticity was estimated as the capacity of tadpoles to adjust their antipredatory behaviour upon learning to recognize the novel predator. Since reduced activity is a common response to predation risk by larval amphibians, including *P. cultripes*^41, 42^, a reduction in tadpoles’ activity in the presence of predator cues was considered indicative of predator recognition. Hence, we compared the innate responses of tadpoles to water-borne chemical cues from the crayfish with their learning-mediated responses after conditioning by pairing crayfish cues with conspecific alarm cues. A total of 404 tadpoles were tested. A group of tadpoles from each population (*n* = 30–35) were randomly assigned to the ‘conditioned’ treatment and another group (*n* = 31–36) to the ‘non-conditioned’ treatment. Tadpoles in the ‘conditioned’ treatment were exposed to predator cues from crayfish coupled with conspecific alarm cues to induce conditioning, whereas tadpoles in the ‘non-conditioned’ treatment were exposed to predator cues alone. Thus, 10 mL test solution of crayfish cues plus 10 mL test solution of tadpole alarm cues were added in each 3 L housing bucket of tadpoles in the ‘conditioned’ treatment, whereas 10 mL test solution of crayfish cues plus 10 mL test solution of clean water were added in each 3 L housing bucket of tadpoles in the ‘non-conditioned’ treatment. Tadpoles were left undisturbed in their housing buckets overnight, and the next day, we tested basal activity levels of each tadpole both in clean water and in water with crayfish cues, in random order. Tadpoles were tested individually in grey, U-shaped, gutters (101 × 11.4 × 6.4 cm), sealed at both ends with plastic caps, and divided into five parts of equal surface by tracing four crossing lines inside. Batches of fifteen tadpoles (fifteen gutters) were tested at the same time. For the trials, gutters were filled with 3 L of carbon-filtered dechlorinated tap water, and we added 10 mL test solutions of clean water or crayfish cues to each end of each gutter (two frozen aliquots per gutter). We waited 5 min for the aliquots to thaw completely, and then placed a single tadpole in the middle of each gutter, waiting another 5 min before the trials began to allow tadpoles to acclimate. Tadpoles were monitored for 30 min, using the instantaneous scan sampling method, and recording every 1 min the quadrant that each tadpole occupied in the gutter (30 scans per tadpole in total). Activity levels were calculated from the number of lines crossed by each tadpole during each trial^41, 42^. Lack of response to crayfish cues by non-conditioned (naïve) tadpoles indicates lack of innate recognition. Then, if conditioned tadpoles significantly reduce their activity in the presence of crayfish cues, it indicates learned predator cue recognition. Change in tadpole activity in the presence of crayfish cues compared to clean water across ‘non-conditioned’ and ‘conditioned’ treatments was taken as a measure of learning-mediated behavioural plasticity or reaction norm for each population.

We analysed differences between conditioned and non-conditioned tadpoles in their responses to water-borne cues from the alien crayfish and compared innate and learning-mediated behavioural plasticity (i.e. reaction norms) of populations between regions by performing linear models with activity level (i.e. the number of lines crossed by tadpoles over the total observation time) as dependent variable, region, population nested in region, and conditioning treatment (‘conditioned’ vs. ‘non-conditioned’) as three between-subject factors, and the experimental chemical stimulus (‘clean water’ vs. ‘crayfish cues’) as a within-subject factor. Since central and southern populations formed two distinct genetic clusters (see below), and because the two regions were not analogous in their patterns of abundance and history of exposure to the invasive crayfish, we also analysed the responses of tadpoles from both regions separately. Finally, we analysed the effect of conditioning within each population by performing linear models with activity level as dependent variable, conditioning treatment as a between-subject factor, and the experimental chemical stimulus as a within-subject factor. Data normality was verified by Kolmogorov–Smirnov test, and Levene’s test indicated homoscedasticity. Post-hoc pairwise comparisons were calculated using protected Fisher’s LSD tests^76^. Analyses were performed using Statistica v12.0 (StatSoft Inc., Tulsa, OK, USA).

### Crayfish abundance

During springs 2017 and 2018, we sampled the study populations repeatedly (6–10 times each) using fyke-nets to measure abundance of red swamp crayfish. Unbaited nets were set underwater in the ponds at twilight and removed the next morning. Number of traps in the populations was adjusted according to pond size, and traps were distributed in the ponds homogeneously. Sampling effort in each population was calculated as the total number of traps multiplied by total sampling duration (i.e. total number of hours that all the fyke-nets were underwater). The number of crayfish of different size (carapace length) classes captured in each population was registered every sampling day. Then, we calculated crayfish density in each population as the total number of crayfish of each size class divided by the surface area of the pond and corrected by the sampling effort. Captured crayfish were removed from the ponds and euthanized following the AVMA Guidelines for invasive species^77^.

### Molecular analyses

We assessed the pattern of genetic diversity of the study populations within and across regions through nuclear DNA microsatellite analysis from tissue samples of tadpoles. Thirty tadpoles (Gosner stage 25^71^) were collected in each population. A portion of the tail tip (the last 5 mm) was clipped, and we immediately released them back in the field. Tail clips were introduced in individualized tagged tubes with 70% ethanol and stored at − 20 °C for DNA extraction and genotyping.

Genomic DNA was extracted from tissue samples using a modified high-salt DNA extraction method^78, 79^. Twelve microsatellite loci from *P. cultripes* (Pc 3.1, Pc 3.2, Pc 3.23, Pc 3.24, Pc 3.25, Pc 3.4, Pc 3.7, Pc 3.9, Pc 4.1, Pc 4.4, Pc 4.5, Pc 4.9)^80^ were multiplexed in three polymerase chain reactions (PCRs), with forward primers labelled with fluorescent dyes. PCR reactions were performed using Type-it Microsatellite PCR kits (Qiagen), in a total volume of 15 μL, containing 7.5 μL of master mix, 1.2 μL of primer mix (0.12–0.4 μM of each primer), and RNase-free H_2_O up to 15 μL. PCR thermal cycling conditions were as follows: initial denaturation at 95 °C for 5 min; followed by 30 cycles of denaturation at 95 °C for 30 s, annealing at 60 °C for 90 s, extension at 72 °C for 30 s; and a final extension step of 30 min at 60 °C. PCR products were analysed by agarose gel electrophoresis (2%) on a 3130XL Genetic Analyzer (Applied Biosystems, Foster City, CA, USA), and fragments were scored using GeneMapper v5.0 (Applied Biosystems, Foster City, CA, USA).

Presence of null alleles was assessed with Micro-Checker v2.2.3^81^, using a 99% confidence interval and 1000 randomizations. To check for deviations from Hardy–Weinberg equilibrium (HWE) and evidence of linkage disequilibrium (LD), we performed Fisher’s exact test on multilocus genotypes using Genepop v4.7.0^82^, with 10 000 dememorization steps, 1000 batches and 10 000 iterations per batch. A sequential Bonferroni correction for multiple testing was applied^83^.

We estimated allelic richness (*A*), number of effective alleles (*A*_*e*_), Shannon information index (*I*), observed (*H*_*o*_) and expected (*H*_*e*_) heterozygosity, unbiased expected heterozygosity (*uH*_*e*_), and the inbreeding coefficient –expressed as Wright’s fixation index (*F*_*IS*_)– for each locus and population using GenAlEx v6.51b2^84^. Comparisons between pairs of populations were performed with the G-statistic subroutine, applying a sequential Bonferroni correction to test for significance. We performed Principal Coordinate Analysis (PCoA) based on pairwise Hedrick’s *G*_*ST*_, Jost’s *D* and Nei’s genetic distance matrices among populations. To test for isolation by distance (IBD), we used paired Mantel tests assessing the correlation between genetic distance matrices and the geographic distance matrix. Genetic variation within and among populations was determined using Analysis of Molecular Variance (AMOVA) with 999 permutations. We also estimated the effective number of breeders (*N*_*b*_) in the populations using the sibship frequency method implemented in Colony v2.0.6.8^85^. Analyses were performed by assuming polygamy in both sexes^86^, with “very high” likelihood precision, “very long” run length and using no sibship prior. We also explored the probability of siblings, and we obtained similar results to our expectations: tadpoles come from between 5–9 egg clutches in every population, with some degree of polygamy.

To characterize population genetic structure across study populations we performed unsupervised Bayesian clustering analyses using Structure v2.3.4^87^. The number of clusters (*K*) best explaining the genetic data was determined by performing ten replicates for each *K* value (1–10), using an admixture model with correlated allele frequencies and 50 000 burn-in and 50 000 post burn-in iteration steps^87, 88^. We summarized clustering results using the online version of Clumpak^89^ and explored the relative likelihood of different *K* values using the Δ*K* (‘Evanno’) method^90^ in Structure Harvester v0.6.94^91^.

## Results

### Behavioural plasticity

**Figure 1:**
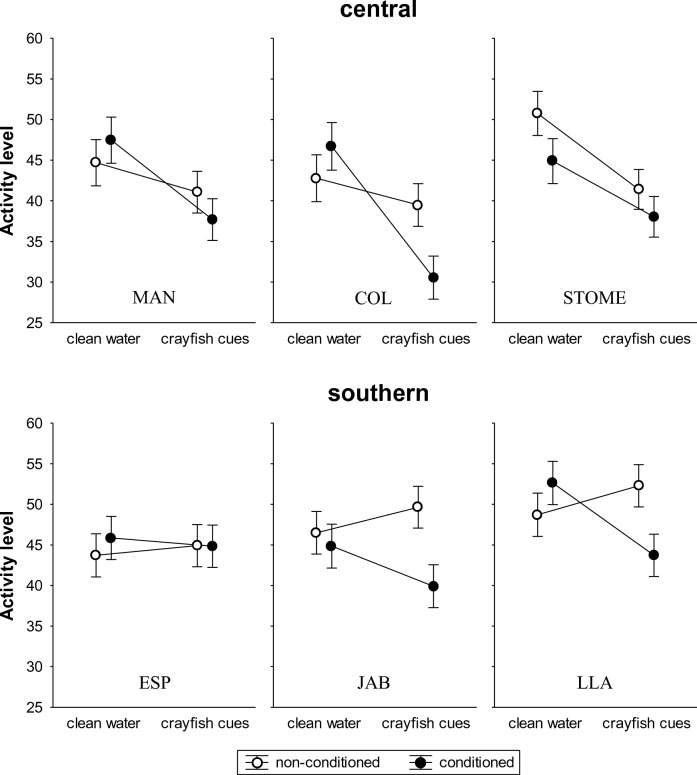
Activity levels (mean ± SE number of lines crossed during 30 min) of non-conditioned (open circles) and conditioned (solid circles) *Pelobates cultripes* tadpoles, either in clean water or in the presence of chemical cues from invasive crayfish, in six populations from two different regions of the Iberian Peninsula, central and southern Spain. *MAN *Manzanares, *COL* Colmenar, *STOME* Sto. Tomé, *ESP* Espajosas, *JAB *Jabata, *LLA *El Llano.

Overall activity of tadpoles was significantly higher in the southern than in the central region (mean ± SE = 46.5 ± 0.9 vs. 42.1 ± 0.9 number of lines crossed), but did not differ across populations (Table 1; Fig. 1). Activity levels of non-conditioned and conditioned tadpoles did not significantly differ overall, and the interactions between conditioning treatment and region or population nested in region were not significant (Table 1). However, we found a significant interaction between conditioning treatment and testing predator cues (Table 1). Behavioural plasticity did not significantly differ between regions (i.e., the interaction between region, conditioning treatment and testing predator cues was not significant), but it did across populations (i.e., the interaction between population nested in region, conditioning treatment and testing predator cues was significant) (Table 1). Behavioural plasticity significantly differed across central populations, but not across southern populations (Table 1). In central Spain, reaction norms were similar between MAN and COL and between MAN and STOME, but differed significantly between COL and STOME (Table 1; Fig. 1), the latter of which lacks crayfish.

**Table 1:** Results of the linear models analysing the effects of conditioning treatment (‘conditioned’ vs. ‘non-conditioned’) and testing predator cues (‘clean water’ vs. ‘crayfish cues’) on activity levels of tadpoles, in six *Pelobates cultripes* populations from two different regions of the Iberian Peninsula, central and southern Spain.﻿﻿ *MAN* Manzanares, *COL* Colmenar, *STOME* Sto. Tomé, *ESP* Espajosas, *JAB* Jabata, *LLA* El Llano.

Effect	*df*	*F*	*p*
Region	1.392	11.17	< 0.001
Population (region)	4.392	2.04	0.09
Conditioning treatment	1.392	3.42	0.07
Region × conditioning treatment	1.392	0.01	0.96
Population (region) × conditioning treatment	4.392	0.81	0.52
Conditioning treatment × testing predator cues	1.392	15.66	< 0.0001
Region × conditioning treatment × testing predator cues	1.392	0.40	0.53
Population(region) × conditioning treatment × testing predator cues	4.392	2.59	0.04
Central			
Population × conditioning treatment × testing predator cues	2.188	3.1	0.04
Population (MAN vs. COL) × conditioning treatment × testing predator cues	1.121	0.96	0.33
Population (MAN vs. STOME) × conditioning treatment × testing predator cues	1.129	2.25	0.14
Population (COL vs. STOME) × conditioning treatment × testing predator cues	1.126	6.46	0.01
MAN			
Conditioning treatment × testing predator cues	1.62	1.77	0.19
Testing predator cues	1.62	8.37	0.005
COL			
Conditioning treatment × testing predator cues	1.59	6.46	0.01
STOME			
Conditioning treatment × testing predator cues	1.67	0.50	0.48
Testing predator cues	1.67	21.34	< 0.0001
Southern			
Population × conditioning treatment × testing predator cues	2.204	1.90	0.15
ESP			
Conditioning treatment × testing predator cues	1.68	0.37	0.55
Testing predator cues	1.68	0.01	0.96
JAB			
Conditioning treatment × testing predator cues	1.68	4.08	0.04
LLA			
Conditioning treatment × testing predator cues	1.68	12.61	< 0.001

**Figure 2:**
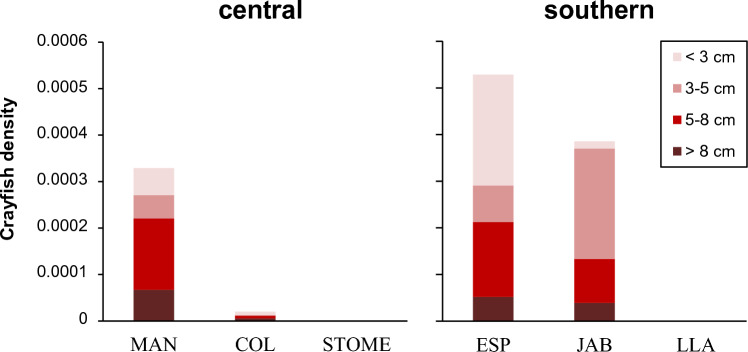
Abundance of crayfish (total individuals/m^2^/h) of different carapace length classes estimated in the populations. *MAN *Manzanares, *COL* Colmenar, *STOME* Sto. Tomé, *ESP* Espajosas, *JAB* Jabata, *LLA* El Llano.

Within the central region, the interaction between conditioning treatment and testing predator cues was not significant in MAN or STOME (Table 1; Fig. 1). In both populations, activity of tadpoles (conditioned and non-conditioned pooled) was reduced in water with crayfish cues with respect to clean water (i.e., the effect of testing predator cues was significant; Table 1), thus indicating innate recognition of these cues as a threat (Fig. 1). In COL, the interaction between conditioning treatment and testing predator cues was significant (Table 1; Fig. 1). Activity of non-conditioned tadpoles in the presence of crayfish cues did not change with respect to their activity in clean water (Fisher’s LSD, *p* = 0.36), indicating lack of innate recognition of chemical cues from the invasive crayfish. However, tadpoles conditioned with conspecific alarm cues in combination with crayfish cues significantly reduced their activity in the presence of crayfish cues compared to clean water (*p* < 0.0001), indicating learned cue recognition of the crayfish in this population (Fig. 1). Within the southern region, the interaction between conditioning treatment and testing predator cues was not significant in ESP (Table 1; Fig. 1), and activity of tadpoles (conditioned and non-conditioned pooled) was similar in the presence of crayfish cues and in clean water, (i.e., the effect of testing predator cues was not significant; Table 1), thus indicating lack of response –innate or learned– to crayfish cues in this population (Fig. 1). In contrast, the interaction between conditioning treatment and testing predator cues was significant in JAB and LLA (Table 1; Fig. 1). Activity of non-conditioned tadpoles was similar in the presence of crayfish cues and in clean water (Fisher’s LSD, JAB: *p* = 0.26; LLA: *p* = 0.16), indicating lack of innate recognition of crayfish cues in both populations (Fig. 1). In JAB, conditioned tadpoles reduced their activity in the presence of crayfish cues compared to clean water, although this reduction did not reach statistical significance (*p* = 0.09). However, activity of conditioned tadpoles was significantly lower than activity of non-conditioned tadpoles in water with crayfish cues (*p* = 0.01), suggesting some degree of learned cue recognition of the invasive crayfish in this population (Fig. 1). Conditioned tadpoles from LLA significantly reduced their activity in the presence of crayfish cues compared to clean water (*p* < 0.001), indicating learned cue recognition of the invasive predator in this population (Fig. 1). Behavioural plasticity of tadpoles was not associated with the abundance of crayfish estimated in the populations (Fig. 2).

### Population genetics

There were significant deviations from Hardy–Weinberg equilibrium (HWE) associated with an excess of heterozygotes in loci Pc4.1, Pc4.4 and Pc4.5 in MAN, and locus Pc3.4 in LLA. Loci Pc3.24 and Pc3.1 presented an excess of homozygotes in ESP, likely due to the existence of null alleles. We also found evidence of linkage disequilibrium (LD) between loci Pc3.1–Pc4.5 in MAN and Pc3.2–Pc4.4 in LLA. Since disequilibria occurred in different populations, these loci were not discarded for subsequent analyses.

Analysis of microsatellite loci showed strong differences across regions in genetic diversity of the populations. Southern populations showed higher allelic richness, number of effective alleles, Shannon information index, and heterozygosity than the central populations, which in turn presented higher *F*_*IS*_ values (Table 2). Allelic richness and heterozygosity were low to moderate, with scarce or inexistent inbreeding (*H*_*o*_ > *H*_*e*_, except for ESP and LLA). Estimates of effective number of breeders were similar between central and southern populations, ranging from 9 to 17 reproductive individuals (Table 2). Within regions, genetic differentiation among southern populations was very low, whereas higher genetic divergence was observed among central populations (Table 3). IBD was not significant among central (Mantel test, Hedrick’s* G*_*ST*_: *R*_*xy*_ = 0.86, *p* = 0.36; Jost’s *D*: *R*_*xy*_ = 0.83, *p* = 0.33; Nei’s:* R*_*xy*_ = 0.81, *p* = 0.3) or southern (Mantel test, Hedrick’s* G*_*ST*_: *R*_*xy*_ = 0.97, *p* = 0.15; Jost’s *D*: *R*_*xy*_ = 0.94, *p* = 0.16; Nei’s: *R*_*xy*_ = 0.9, *p* = 0.17) populations (Table 3).

**Table 2:** Genetic diversity in six *Pelobates cultripes* populations from two different regions of the Iberian Peninsula, central and southern Spain.

	*A*	*A*_*e*_	*I*	*H*_*o*_	*H*_*e*_	*uH*_*e*_	*F*_*IS*_	*N*_*b*_
	Mean (SE)	Mean (SE)	Mean (SE)	Mean (SE)	Mean (SE)	Mean (SE)	Mean (SE)	(95% CI)
Central								
MAN	2.417 (0.260)	1.884 (0.233)	0.631 (0.109)	0.531 (0.095)	0.394 (0.062)	0.400 (0.064)	− 0.309 (0.079)	11 (6–26)
COL	3.583 (0.379)	1.987 (0.216)	0.757 (0.119)	0.442 (0.072)	0.424 (0.066)	0.431 (0.067)	− 0.042 (0.045)	9 (5–24)
STOME	2.667 (0.466)	1.603 (0.174)	0.528 (0.125)	0.319 (0.076)	0.296 (0.069)	0.301 (0.070)	− 0.077 (0.034)	16 (8–35)
Southern								
ESP	5.333 (0.632)	2.859 (0.407)	1.142 (0.133)	0.564 (0.066)	0.569 (0.056)	0.578 (0.057)	0.030 (0.051)	13 (7–30)
JAB	5.083 (0.793)	2.641 (0.291)	1.097 (0.128)	0.572 (0.059)	0.559 (0.054)	0.568 (0.055)	− 0.018 (0.033)	17 (9–34)
LLA	4.833 (0.613)	2.623 (0.327)	1.038 (0.148)	0.511 (0.070)	0.527 (0.072)	0.536 (0.073)	0.020 (0.040)	9 (5–24)

*A* number of alleles, *A*_*e*_ number of effective alleles, *I* Shannon information index, *H*_*o*_ observed heterozygosity, *H*_*e*_ expected heterozygosity, *uH*_*e*_ unbiased expected heterozygosity, *F*_*IS*_ Wright’s inbreeding coefficient, *N*_*b*_ estimates of effective number of breeders. *MAN *Manzanares, *COL *Colmenar, *STOME *Sto. Tomé, *ESP *Espajosas, *JAB *Jabata, *LLA *El Llano.

**Table 3:** Pairwise values of (**a**) Hedrick’s *G*_*ST*_ (lower triangular matrix) and Jost’s *D* (upper triangular matrix) and (**b**) Nei’s (lower triangular matrix) and geographical distance (upper triangular matrix; km) among six *Pelobates cultripes* populations from two different regions of the Iberian Peninsula, central and southern Spain.

	Central			Southern		
	MAN	COL	STOME	ESP	JAB	LLA
**(a)**						
MAN		0.087	0.246	0.282	0.270	0.267
COL	0.058		0.161	0.228	0.226	0.226
STOME	0.186	0.122		0.276	0.277	0.271
ESP	0.129	0.101	0.149		0.022	0.037
JAB	0.126	0.101	0.153	0.008		0.029
LLA	0.132	0.108	0.158	0.015	0.012	
**(b)**						
MAN		6	58			
COL	0.100		60			
STOME	0.286	0.180				
ESP	0.330	0.265	0.306		6	31
JAB	0.316	0.263	0.310	0.044		25
LLA	0.316	0.267	0.308	0.059	0.049	

*MAN* Manzanares, *COL *Colmenar, *STOME *Sto. Tomé, *ESP *Espajosas, *JAB *Jabata, *LLA *El Llano.

Partitioning of overall genetic variation within and among populations by AMOVA indicated that 82% of genetic variation was distributed within populations, whereas 18% was distributed among populations (AMOVA, *F*_*ST*_ = 0.18, *p* = 0.001; *F*_*IS*_ =  − 0.04, *p* = 0.99; *F*_*IT*_ = 0.15, *p* = 0.001). Separated analyses by region yielded similar results for central populations (81% within vs. 19% among populations; AMOVA, *F*_*ST*_ = 0.22, *p* = 0.001; *F*_*IS*_ =  − 0.14, *p* = 0.99; *F*_*IT*_ = 0.11, *p* = 0.001), but indicated much lower genetic structure among southern populations (96% within vs. 2% among populations; AMOVA, *F*_*ST*_ = 0.02, *p* = 0.001; *F*_*IS*_ = 0.02, *p* = 0.17; *F*_*IT*_ = 0.04, *p* = 0.02).

**Figure 3:**
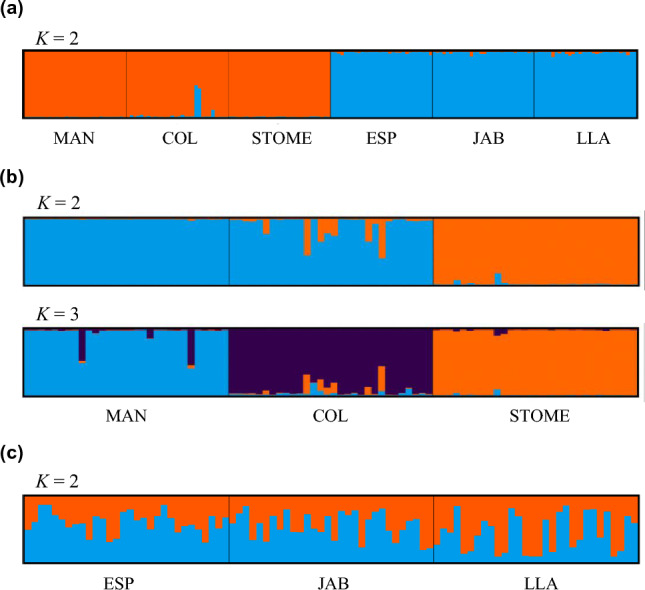
Summary results of the Bayesian clustering assignment implemented in Structure, with colours indicating clusters, or populations. (**a**) Number of *K* clusters best explaining genetic data of the six *Pelobates cultripes* populations from two different regions of the Iberian Peninsula, central and southern Spain. (**b**) Separated analysis for central region. Optimal number of clusters was *K* = 2 (upper panel), closely followed by *K* = 3 (lower panel). (**c**) Separated analysis for southern region. *MAN *Manzanares, *COL *Colmenar, *STOME *Sto. Tomé, *ESP* Espajosas, *JAB *Jabata, *LLA *El Llano.

Number of clusters best explaining genetic data according to Evanno method (Δ*K*) was *K* = 2, simply corresponding to the central and southern regions (Fig. 3a). When performing separated analyses by region, the optimal number of clusters in the central region was *K* = 2, with STOME clearly separated from the other two central populations, but closely followed by *K* = 3, grouping individuals into three separate populations (Fig. 3b). In the southern region, the optimal number of clusters was *K* = 2, with individuals admixed at approximately the same proportions (Fig. 3c).

## Discussion

Our findings demonstrate widespread learning abilities of spadefoot toad tadpoles to recognize and respond to water-borne cues from a new predator, the invasive crayfish *P. clarkii*. We found lack of innate recognition of crayfish odour in four out of the six populations across the two study regions, and three out of these four were able to recognize it as a threat after conditioning with conspecific alarm cues. The lack of innate recognition of invasive crayfish by Iberian spadefoot toad populations is not unexpected, given that *P. clarkii* was introduced in the Iberian Peninsula ca. 50 years ago, a relatively short evolutionary time for adaptation to occur in temperate amphibians with long generation times (1–2 years). In our previous studies, *P. cultripes* and *P. perezi* tadpoles from Doñana National Park also failed to innately recognize invasive *P. clarkii* chemical cues^38, 41^. Here we extend this observation to other regions of the Iberian Peninsula. Naïve tadpoles of several anuran species, including *P. cultripes* and *P. perezi*, from southern Portugal have been reported to exhibit antipredatory behaviour in response to joint crayfish and attacked conspecific cues^64, 68, 69^, but responses to crayfish cues alone remain to be tested. Recently, Melotto et al.^11^ found that tadpoles of several amphibian species, especially those sharing coevolutionary history with native crayfish, strongly responded to visual cues from *P. clarkii*, whereas their responses to chemical cues from invasive crayfish were weak and contrasting. Altogether, our study reinforces the idea that responses of amphibians to chemical cues from novel exotic predators are largely mediated by consumed conspecifics^68^.

Different histories of coexistence with predators may also lead to dissimilar evolved responses to invasive crayfish among populations. In fact, generalization of predator recognition (i.e. when prey lacking innate recognition of novel predators have the ability to label them as predatory if they are phylogenetically closely related to already known predators^92^) has been demonstrated in larval amphibians^93–95^. In the Iberian Peninsula, the native crayfish *Austropotamobius pallipes* has been historically absent from Doñana area and almost from the entire Madrid region, but this indigenous species was present in the area of STOME population (in the limits between Segovia and Madrid provinces) about 50 years ago, before Iberian crayfish populations were drastically decimated by the crayfish plague caused by the pathogen *Aphanomyces astaci*^96–98^. Interestingly, we observed a significant reduction in activity of naïve tadpoles in response to water-borne cues from *P. clarkii* in the STOME population (Fig. 1). This suggests that a certain degree of generalized predator recognition of the alien crayfish linked to the presence of a native crayfish in the recent past might be taking place in this non-invaded population. However, an innate response to crayfish cues was observed also in the MAN population (Fig. 1), where *A. pallipes* has been historically absent. Although the arrival of *P. clarkii* to central Spain is fairly recent (ca. 35 years), we cannot completely discard that innate recognition of crayfish cues could have evolved in this population under the predatory pressure posed by the alien crayfish during the last decades.

Predator diet seems to be a critical factor in the recognition of alien predators by naïve amphibian larvae, and the consumption of conspecific tadpoles is precisely the ecological scenario in which learned recognition would take place. Even though innate responses may fail to be triggered against novel predators, prey can still rely on learning to reduce their impact. Learning of new threats through cognitive association with alarm cues has been observed in a variety of aquatic prey (see^47^ for an extended review), including amphibians^44–46, 95^. In a previous study, we demonstrated that learning via association with conspecific alarm cues allows successful activation of antipredatory responses by spadefoot toad tadpoles against *P. clarkii*, increasing larval survival in predatory assays^41^. Here we show that such learning ability is common to multiple populations of *P. cultripes* regardless of the intensity of the invasion by crayfish. This widespread learned predator recognition suggests that amphibian larvae might broadly benefit from associative learning to recognize and avoid new predatory threats, thus modulating the initial advantage of alien predators in amphibian assemblages.

According to the ‘plasticity-first’ evolution hypothesis^14–16^, we expected the level of predatory threat posed by the crayfish (i.e. abundance and history of exposure) to be positively associated with learning ability across populations^58–60^. A rapid shift towards learning-mediated antipredator responses might be facilitated by differential survival of individuals prone to learning. In addition, higher levels of predation mean higher concentration of conspecific alarm cues in the environment, presumably resulting in learning reinforcement^43, 99–102^. However, we did not find evidence for correlation between the crayfish abundance and the behavioural plasticity of tadpoles across populations. Reaction norms were similar across southern populations, even though abundance of crayfish was considerably higher in ESP and JAB (Figs. 1 and 2), likely due to higher gene flow and lack of genetic substructuring. Further, the separated analysis for ESP indicated lack of learned response to crayfish cues in this population. Among central populations, behavioural plasticity of tadpoles differed significantly between the non-invaded STOME and the intermediate COL, but not between STOME and the most heavily invaded MAN (Figs. 1 and 2). Also, the most responsive population (COL) was not the one with the highest abundance of crayfish (MAN). Predator learning therefore does not seem to be associated with history of coexistence with crayfish, suggesting that selection by the invader is likely not the main factor explaining population divergence in learning abilities. Amphibian populations facing *P. clarkii* might be co-opting pre-existing antipredatory adaptations evolved to face current or ancestral predators (such as the native *A. pallipes* in STOME). Further, the capacity for learning itself may have also evolved under selective pressures imposed by additional factors varying across populations, consequently affecting larval skills to acquire recognition of new predatory threats^103^.

Learning abilities and behavioural plasticity of tadpoles also vary according to genetic divergence. The similar behavioural responses to *P. clarkii* observed within the southern region may have arisen from a combination of (1) longer history of exposure to this invasive predator –crayfish were firstly introduced in the Guadalquivir marshes, reaching the Spanish central plateau later– and (2) higher levels of gene flow, as southern populations inhabit a highly interconnected pond network. In contrast, a lower connectivity and a higher genetic differentiation seem to be responsible for the more divergent plastic responses found among the central populations. In consequence, these more isolated populations may be at a greater risk from invasive predatory crayfish.

Our results emphasize the importance of integrating phenotypic plasticity and behavior of native organisms in the management of biological invasions, in order to achieve the conservation goal of long-term persistence. Indeed, the capacity to plastically modify behavior in response to new or unusual challenges (i.e., the cognitive buffer^104^) is an essential mechanism for animals to cope with rapid environmental changes such as the introduction of novel predators. By enabling tadpoles to adjust defensive behaviour and activate inducible defences, widespread learned predator recognition may be decisive for amphibian populations to persist in the new ecological context posed by aliens, soothing the impact of invasions and buying time for innate recognition to evolve^17, 18^.

## Data Availability

Data supporting the findings are available from Dryad Digital repository (https://datadryad.org/stash/share/OiStTZZeDejQEnu0VF9gm-hkI1Rwa0J4b2yLeiPi9-k).
